# Mental Health Disorders and Associated Risk Factors in Quarantined Adults During the COVID-19 Outbreak in China: Cross-Sectional Study

**DOI:** 10.2196/20328

**Published:** 2020-08-06

**Authors:** Yan Guo, Chao Cheng, Yu Zeng, Yiran Li, Mengting Zhu, Weixiong Yang, He Xu, Xiaohua Li, Jinhang Leng, Aliza Monroe-Wise, Shaomin Wu

**Affiliations:** 1 Department of Medical Statistics School of Public Health Sun Yat-sen University Guangzhou China; 2 Sun Yat-sen Center for Migrant Health Policy Guangzhou China; 3 Sun Yat-sen Center for Global Health Guangzhou China; 4 Department of Thoracic Surgery The First Affiliated Hospital of Sun Yat-sen University Guangzhou China; 5 Department of Global Health University of Washington Seattle, WA United States

**Keywords:** COVID-19, anxiety or depressive symptoms, quarantine, risk and protective factors

## Abstract

**Background:**

People undergoing mass home- and community-based quarantine are vulnerable to mental health disorders during outbreaks of coronavirus disease (COVID-19), but few studies have evaluated the associated psychosocial factors.

**Objective:**

This study aimed to estimate the prevalence of anxiety and depressive symptoms and identify associated demographic and psychosocial factors in the general Chinese population during the COVID-19 pandemic quarantine period.

**Methods:**

Participants aged 18 years or above were recruited in a cross-sectional online survey using snowball sampling from February 26-29, 2020. The survey included questions on demographics, family relationships, chronic diseases, quarantine conditions, lifestyle, COVID-19 infection, and anxiety and depressive symptoms. Logistic regression analyses were conducted to identify factors associated with elevated anxiety or depressive symptoms.

**Results:**

Out of 2331 participants, 762 (32.7%) experienced elevated anxiety or depressive symptoms. Nine risk factors associated with anxiety or depressive symptoms included younger age, reduced income, having cancer or other chronic diseases, having family members living with cancer, concerns related to COVID-19 infection for themselves or family members, living alone, having family conflicts, having <3 or >8 hours of sedentary time per day, and worsened sleep quality.

**Conclusions:**

The findings highlight an urgent need for psychological support for populations at high risk for elevated anxiety or depressive symptoms during the COVID-19 pandemic.

## Introduction

Pandemics and other public health crises such as severe acute respiratory syndrome (SARS), Ebola, and the current coronavirus disease (COVID-19) pandemic often result in elevated rates of mental health problems [1-3]. Anxiety, depressive symptoms, and posttraumatic stress disorder (PTSD) are the most common mental health problems during pandemics [4]. To fight against the COVID-19 pandemic, quarantine strategies have been implemented in many countries including China [5,6]. However, large-scale and long-term quarantine may have negative impacts on people’s mental health. A recent study found that the prevalence of depressive symptoms was 20.1% in the Chinese population during the first month of widely implemented quarantine due to COVID-19 [7], which is much higher than previous reports of the lifetime rate of depressive symptoms in this population (6.8%), based on a representative sample [8]. However, empirical evidence regarding psychological disorders and related risk factors among people undergoing mass home- and community-based quarantine are still lacking.

Although several studies have reported on factors associated with mental health disorders during the COVID-19 pandemic, some factors particularly characteristic of the pandemic and corresponding quarantine strategy remain understudied. In the existing literature, factors such as demographic characteristics, lifestyle, and concerns about COVID-19 infection have been examined during the pandemic [7,9,10]. Specifically, reduced income, increased sedentary time, poor sleep quality, and concerns for COVID-19 infection were reported as risk factors for elevated anxiety or depressive symptoms [7,9,10]. Yet little attention has been paid to social factors such as an individual’s household composition (living alone or with others) and family relationships, all of which may reflect people’s social connectedness and affect their psychological well-being especially under social distancing or quarantine policies. During the COVID-19 quarantine period, a strict movement restriction was widely implemented in China [6]. People either spent more time with family members at home or experienced isolation during quarantine. The former scenario could lead to increased family conflicts during this hardship, and the latter may result in social isolation [11]. However, the potential risk factors for mental health symptoms arising from the COVID-19 pandemic have rarely been studied during the quarantine.

In addition, previous studies have shown mixed findings about the effects of some factors such as age on anxiety or depressive symptoms during the COVID-19 pandemic. One study reported that individuals between 18 and 30 years or above 60 years had the highest rates of psychological distress during the COVID-19 outbreaks [12], while another study found only younger people presented higher levels of stress and anxiety [13]. Two other studies found no association between age and mental health problems during the COVID-19 outbreaks [10,14].

Some populations such as people living with cancer or other chronic diseases may be at increased risk for developing mental health problems during a pandemic due to their tenuous physical health, barriers to accessing medical treatment, higher risks of COVID-19 infection, and higher probability of severe illness if infected [15-19]. During the COVID-19 quarantine period, patients with cancer or other chronic diseases may experience increased challenges related to receiving routine medical care due to mobility restrictions and the potential shortages of medical workers and essential medicines [20]. One study found that the likelihood of COVID-19 infection in cancer patients was twice as high as that in the general population [17]. In addition, the World Health Organization (WHO) reported that patients with pre-existing noncommunicable diseases, including cardiovascular disease, chronic respiratory disease, diabetes, and cancer, are at increased risk of severe illness from COVID-19 [21]. Therefore, the WHO has urged the global medical community to pay additional attention to mental health in this vulnerable population, especially during the pandemic [21]. Yet, studies on mental health problems in cancer patients or other chronic diseases are scarce.

To fill these gaps in the literature, the current study aimed to estimate the prevalence of anxiety or depressive symptoms and identify associated demographic and psychosocial factors in the general Chinese population during the COVID-19 pandemic quarantine in China in February 2020. Specifically, we examined the effects of associated factors including demographics, family relationships, chronic disease status, quarantine conditions, lifestyle, and COVID-19 infection. Special attention was paid to characteristics particularly relevant to the COVID-19 pandemic quarantine such as household composition, family conflict, and chronic disease status.

## Methods

Participants aged 18 years or above were recruited through a snowball sampling process via WeChat from February 26-29, 2020 during the community- or home-based quarantine in China. WeChat is the most widely used social media platform in China with over 1 billion active users [22]. We developed an online questionnaire using Questionnaire Star, the link to which could be shared via WeChat. Clicking the survey link in WeChat took participants directly to the online questionnaire. The online survey link was initially and purposely sent to 10 participants who were chosen to ensure a broad representation of age, gender, educational level, and chronic diseases status (eg, with or without chronic diseases). We asked these participants to send the survey link to friends on their WeChat contact list whom they considered suitable for this survey, and their friends were also encouraged to send the link to their own WeChat contact networks. The snowball sampling process continued until a sufficient sample size was reached. To recruit cancer patients and their family members, oncologists sent the survey link to patient groups in WeChat and encouraged them to participate in the study. Participants anonymously completed the self-administered electronic questionnaire for about 15 minutes with no financial incentive. Participants were allowed to reaccess the survey link but doing so erased their previous data. Although the probability of repeated completion by the same participants cannot be ruled out, such instances were rare as they were asked to complete it once and there was no incentive for repeat submissions.

Data on demographics (eg, age, gender, marital status, educational status, household composition, individual income), family relationships (eg, family conflict), chronic diseases (eg, cancer, hypertension, diabetes, asthma, cerebrovascular or cardiovascular diseases), quarantine conditions (eg, quarantine duration, frequency of going out during the quarantine), lifestyle (eg, physical activity, sedentary behavior, sleep quality), COVID-19 infection (eg, confirmed or suspected cases among family members, friends, colleagues, or in the community), and anxiety and depressive symptoms were collected.

Anxiety and depressive symptoms were assessed by the 14-item Hospital Anxiety and Depression Scale (HADS) that comprises two 7-item subscales; participants with an Anxiety subscale (HADS-A) score ≥8 or a Depression subscale (HADS-D) score ≥8 were classified as having elevated anxiety or depressive symptoms [23]. We chose HADS because not only does it measure anxiety and depressive symptoms simultaneously with good reliability and validity [24], but it is also easy to understand and brief, requiring minimal time to complete. In comparison, other tools such as the Center for Epidemiologic Studies Depression Scale (CESD) and the Patient Health Questionnaire-9 (PHQ-9) measure only depressive symptoms. In addition, the 20-item CESD is too long to complete, whereas PHQ-9 has one item that is particularly sensitive and unsuitable for the general population during the stressful time of the pandemic (ie, “Thoughts that you would be better off dead, or thoughts of hurting yourself in some way”) [24]. Therefore, based on the feedback from the pilot study prior to the large-scale survey deployment, we chose to use HADS.

The sample size was calculated based on the estimated rate of anxiety or depressive symptoms in the general population. A pre–COVID-19 epidemiological study reported the lifetime prevalence of anxiety and depressive symptoms in the Chinese population as 7.6% and 6.8%, respectively [8]. Considering the likelihood of increased rates of anxiety and depressive symptoms during pandemics, we hypothesized a rate of 18% for anxiety or depressive symptoms in the general population. With a significance level set at .05, an absolute error of 1.8%, and allowing for up to 10% invalid questionnaires, we estimated a minimum sample size of 2004 for the current study.

Logistic regression models were employed to identify factors associated with elevated anxiety or depressive symptoms. All variables were categorized and described by frequencies and percentages. Univariate logistic regression models were used to analyze the distribution of anxiety or depressive symptoms among different categories for each variable and select risk factors for multivariate analysis. For example, to identify whether chronic disease status was a potential risk factor for anxiety or depressive symptoms, we compared the rate of anxiety or depressive symptoms among participants with chronic diseases to that of participants without. Variables with *P*<.10 in univariate analysis were included in the multivariate logistic regression model and those with *P*<.05 were retained in the final model. R software version 3.5.1 (R foundation for Statistical Computing) was used for data analyses.

This study was approved by the institutional review board of the School of Public Health, Sun Yat-sen University, and all participants provided informed consent.

## Results

Out of 2441 questionnaires collected, 2331 (95.5%) were deemed valid using a quality-control question, which stated that its purpose was to screen out invalid questionnaires and requested participants to check a specific option. Questionnaires that checked the correct option were considered valid. The mean age of participants was 34.4 (SD 11.1) years, 56.1% (n=1307) were female, 60.0% (n=1398) were married, 73.7% (n=1718) had a bachelor’s degree or above, 11.5% (n=269) had a chronic disease, 41.6%(n=970) had family members with a chronic disease, 44.7% (n=1041) had been quarantined for over 3 weeks, and 54.5% (n=1271) went out no more than once a week. In total, 32.7% (n=762) of participants experienced elevated anxiety or depressive symptoms. Specifically, 25.4% (n=592) experienced anxiety, 21.3% (n=496) experienced depressive symptoms, and 13.9% (n=326) experienced both anxiety and depressive symptoms.

Rates of elevated anxiety or depressive symptoms among people without chronic diseases, with cancer, and with other chronic diseases were 31.5% (n=649), 34.8% (n=32), and 45.8% (n=81), respectively. Rates of elevated anxiety or depressive symptoms among people whose family members had no chronic diseases, cancer, and other chronic diseases were 31.7% (n=431), 45.0% (n=36), and 33.1% (n=295), respectively.

As shown in Table 1, nine variables were retained in the multivariate logistic regression model, and the odds ratio (OR) of each variable was adjusted by the remaining eight variables in the model. Risk factors associated with elevated anxiety or depressive symptoms included younger age (<40 years) (41-55 years: OR 0.666, 95% CI 0.494-0.899; ≥56 years: OR 0.507, 95% CI 0.304-0.847), reduced income during quarantine (OR 1.441, 95% CI 1.179-1.760), having cancer (OR 1.900, 95% CI 1.146-3.149) or other chronic diseases (OR 2.222, 95% CI 0.304-0.847), having family members living with cancer (OR 1.821, 95% CI 1.119-2.964), concerns for COVID-19 infection for themselves or family members (moderate concern: OR 1.603, 95% CI 1.216-2.115; severe concern: OR 2.315, 95% CI 1.705-3.142), living alone (living with family or others: OR 0.595, 95% CI 0.422-0.839), having family conflicts during the COVID-19 outbreak (OR 1.707, 95% CI 1.267-2.299), having <3 (OR 1.702, 95% CI 1.407-2.059) or >8 (OR 1.458, 95% CI 1.193-1.783) hours of sedentary time per day, and worsened sleep quality (OR 2.917, 95% CI 2.319-3.670) during the quarantine.

**Table 1:** Logistic regression of associated factors for elevated anxiety or depressive symptoms during the quarantine (N=2331).

Variable				Total, N		Anxiety or depressive symptoms^a^, n (%)		Unadjusted results				Adjusted results		
								Odds ratio (95% CI)		*P* value		Odds ratio (95% CI)		*P* value
**Age (years)**														
	18-25			530		184 (34.7)		1 (ref^b^)		N/A^c^		1 (ref)		N/A
	26-40			1197		406 (33.9)		0.965 (0.778-1.197)		.75		0.836 (0.661-1.058)		.14
	41-55			475		138 (29.1)		0.770 (0.590-1.006)		.06		0.666 (0.494-0.899)		.01
	≥56			129		34 (26.4)		0.673 (0.438-1.035)		.07		0.507 (0.304-0.847)		.01
**Change in income**														
	Unchanged			1571		467 (29.7)		1 (ref)		N/A		1 (ref)		N/A
	Worse			737		290 (39.3)		1.534 (1.277-1.842)		<.001		1.441 (1.179-1.760)		<.001
	Better			23		5 (21.7)		0.657 (0.242-1.779)		.41		0.558 (0.198-1.575)		.27
**Has a chronic disease**														
	None			2062		649 (31.5)		1 (ref)		N/A		1 (ref)		N/A
	Other chronic diseases			177		81 (45.8)		1.837 (1.347-2.505)		<.001		2.222 (1.556-3.172)		<.001
	Cancer			92		32 (34.8)		1.161 (0.749-1.801)		.51		1.900 (1.146-3.149)		.01
**Family members with chronic diseases^d^**														
	None			1361		431 (31.7)		1 (ref)		N/A		1 (ref)		N/A
	Other chronic diseases			890		295 (33.1)		1.070 (0.893-1.281)		.46		0.983 (0.808-1.196)		.86
	Cancer			80		36 (45.0)		1.765 (1.120-2.783)		.01		1.821 (1.119-2.964)		.02
**Concerns about infection for themselves or family members**														
	Little			400		87 (21.8)		1 (ref)		N/A		1 (ref)		N/A
	Moderate			1351		430 (31.8)		1.680 (1.290-2.187)		<.001		1.603 (1.216-2.115)		.001
	Severe			580		245 (42.2)		2.631 (1.971-3.513)		<.001		2.315 (1.705-3.142)		<.001
**Living situation**														
	Alone			168		71 (42.3)		1 (ref)		N/A		1 (ref)		N/A
	With family or others			2163		691 (31.9)		0.641 (0.466-0.882)		.06		0.595 (0.422-0.839)		.003
	**Family conflict^e^**													
		No	1502		409 (27.2)		1 (ref)		N/A		1 (ref)		N/A	
		Yes	829		353 (42.6)		1.982 (1.658-2.369)		<.001		1.702 (1.407-2.059)		<.001	
**Sedentary behavior (hours/day)**														
	3-8			1150		313 (27.2)		1 (ref)		N/A		1 (ref)		N/A
	<3			261		102 (39.1)		1.715 (1.296-2.271)		<.001		1.707 (1.267-2.299)		<.001
	>8			920		347 (37.7)		1.619 (1.344-1.951)		<.001		1.458 (1.193-1.783)		<.001
**Sleep quality during quarantine**														
	Unchanged			1448		384 (26.5)		1 (ref)		N/A		1 (ref)		N/A
	Worse			451		249 (55.2)		3.416 (2.743-4.253)		<.001		2.917 (2.319-3.67)		<.001
	Better			432		129 (29.9)		1.180 (0.931-1.495)		.17		1.043 (0.814-1.335)		.74
**Region**														
	Other provinces			2134		677 (31.7)		1 (ref)		N/A		N/A		N/A
	Hubei Province			197		85 (43.1)		1.633 (1.214-2.197)		.001		N/A		N/A
**Infection cases^f^**														
	None			2285		736 (32.2)		1 (ref)		N/A		N/A		N/A
	Yes			46		26 (56.5)		2.736 (1.517-4.933)		.001		N/A		N/A
**Duration of quarantine**														
	≤3 weeks			1290		414 (32.1)		1 (ref)		N/A		N/A		N/A
	>3 weeks			1041		348 (33.4)		1.063 (0.893-1.264)		.49		N/A		N/A
**Frequency of going out**														
	Never			462		152 (19.9)		1 (ref)		N/A		N/A		N/A
	≤1 time per week			809		256 (33.6)		0.944 (0.740-1.205)		.64		N/A		N/A
	2-4 times per week			769		261 (34.3)		1.048 (0.820-1.339)		.71		N/A		N/A
	≥5 times per week			261		93 (12.2)		0.958 (0.700-1.311)		.79		N/A		N/A

^a^Participants with an HADS-A score ≥8 or an HADS-D score ≥8 were classified as those with elevated anxiety or depressive symptoms.

^b^ref: reference.

^c^N/A: not applicable.

^d^Chronic diseases included the following options: cancer, hypertension, diabetes, cerebrovascular disease (eg, stroke, cerebral infarction, cerebral hemorrhage), ischemic heart disease, chronic hepatitis/cirrhosis, chronic bronchitis/emphysema, asthma, rheumatoid arthritis, psychological disorders, and others specified by the participants.

^e^Family conflict refers to family disputes over COVID-19–related preventive measures (eg, frequency of hand washing), concerns about mutual transmission of coronavirus among family members, and different opinions on information related to COVID-19 (eg, origins of the outbreak) were the three main triggers of family conflict.

^f^Infection cases: whether there were confirmed or suspected cases among family members, friends, colleagues, or in the community.

## Discussion

China’s large-scale quarantine was implemented in late January 2020, resulting in weeks of social isolation. In our sampled population, 32.7% experienced elevated anxiety or depressive symptoms during the quarantine, with 25.4% and 21.3% experiencing anxiety and depressive symptoms, respectively. This is more than triple the rates of lifetime anxiety and depressive symptoms previously reported for the Chinese population (7.6% and 6.8%, respectively) [8]. In our survey, the rates of elevated anxiety or depressive symptoms among participants with cancer or chronic diseases were found to be 34.8% and 45.8%, respectively, which are higher than the rate of 31.5% identified among participants without chronic diseases during the COVID-19 quarantine.

Consistent with the existing literature, this study also found that financial loss, infection concerns, sedentary behavior, poor sleep quality, living with cancer or other chronic diseases, or having family members with cancer were associated with elevated anxiety or depressive symptoms [11,25-27]. Previous studies reported inconsistent results regarding the impact of age on anxiety or depressive symptoms during the COVID-19 outbreak. One study revealed a greater negative psychological impact for both young adults and the elderly [12], while another study found that only younger age was associated with increased stress [13]. In our study, we found that younger people were more likely to experience elevated anxiety or depressive symptoms than those above 40 years. A possible explanation for this result is that younger people might have heavier financial burdens and more access to information about the COVID-19 epidemic through social media, both of which could lead to increased stress [28]. Some studies have indicated that increased sedentary behavior was associated with anxiety and depressive symptoms during the COVID-19 pandemic [29]. Interestingly, our study found associations between both increased (>8 hours per day) and decreased sedentary time (<3 hours per day) and mental health symptoms. This could be explained by people who were working or taking care of sick family members suffering from intensified stress due to the likelihood of exposure to COVID-19. However, this finding needs further exploration.

This study found some unique risk factors associated with elevated anxiety or depressive symptoms in the Chinese population during the COVID-19 outbreak. We found that people who lived alone had an increased risk of anxiety or depressive symptoms, which might be due to diminished social interactions during the quarantine. Conversely, we also found that family conflict related to COVID-19 might be a source of stress contributing to mental health problems. In our survey, the rate of family conflict during the COVID-19 quarantine was found to be 36%, and most family conflict was related to COVID-19, such as disagreements over how family members should protect themselves from the pandemic and different opinions about information related to COVID-19. These results provide important empirical evidence for policy making; tailored strategies are advisable for different populations at high risk of increased psychological distress and problems. Social connectedness and support should be promoted and provided, especially to those who live alone as an effort to protect them from social isolation. Information on COVID-19 and health promotion could be delivered more effectively through multiple channels to minimize family conflict and cultivate better mental health.

There are some limitations in this study. Our sample was more educated than the general Chinese adult population and may not be representative due to limitations of the sampling method used. However, since random sampling is difficult to achieve in a situation like the COVID-19 pandemic, social media–based sampling is a preferred alternative [30]. Though we oversampled cancer patients and their family members through the WeChat contact networks of doctors, the numbers were still small. Findings should be interpreted with caution. Future studies exploring interactions between or among risk factors may be helpful in providing guidance for vulnerable populations.

The results of this study highlight an urgent need for psychological support and counseling for populations at high risk for elevated anxiety or depressive symptoms during the current pandemic or any quarantine implementation. The WHO needs to urge the global medical community to provide screening for mental health problems and psychological services to vulnerable populations such as patients with cancer or other chronic diseases.

## Abbreviations

CESD: Center for Epidemiologic Studies Depression Scale
COVID-19: coronavirus disease
HADS: Hospital Anxiety and Depression Scale
HADS-A: Anxiety subscale of Hospital Anxiety and Depression Scale
HADS-D: Depression subscale of Hospital Anxiety and Depression Scale
PHQ-9: Patient Health Questionnaire-9
PTSD: posttraumatic stress disorder
SARS: severe acute respiratory syndrome
WHO: World Health Organization

## References

[1] Reardon S (2015). Ebola's mental-health wounds linger in Africa. Nature.

[2] Maunder RG (2009). Was SARS a mental health catastrophe?. Gen Hosp Psychiatry.

[3] (2020). Mental health and psychosocial considerations during the COVID-19 outbreak. World Health Organization.

[4] Pfefferbaum B, North CS (2020). Mental Health and the Covid-19 Pandemic. N Engl J Med.

[5] (2020). Coronavirus disease 2019 (COVID-19) Situation Report - 53. World Health Organization.

[6] Guo Y, Li Y, Monroe-Wise A, Yeung SJ, Huang Y (2020). A dynamic residential community-based quarantine strategy: China's experience in fighting COVID-19. Infect Control Hosp Epidemiol.

[7] Huang Y, Zhao N (2020). Generalized anxiety disorder, depressive symptoms and sleep quality during COVID-19 outbreak in China: a web-based cross-sectional survey. Psychiatry Res.

[8] Huang Y, Wang Y, Wang H, Liu Z, Yu X, Yan J, Yu Y, Kou C, Xu X, Lu J, Wang Z, He S, Xu Y, He Y, Li T, Guo W, Tian H, Xu G, Xu X, Ma Y, Wang L, Wang L, Yan Y, Wang B, Xiao S, Zhou L, Li L, Tan L, Zhang T, Ma C, Li Q, Ding H, Geng H, Jia F, Shi J, Wang S, Zhang N, Du X, Du X, Wu Y (2019). Prevalence of mental disorders in China: a cross-sectional epidemiological study. The Lancet Psychiatry.

[9] Pappa S, Ntella V, Giannakas T, Giannakoulis VG, Papoutsi E, Katsaounou P (2020). Prevalence of depression, anxiety, and insomnia among healthcare workers during the COVID-19 pandemic: A systematic review and meta-analysis. Brain Behav Immun.

[10] Wang C, Pan R, Wan X, Tan Y, Xu L, Ho CS, Ho RC (2020). Immediate Psychological Responses and Associated Factors during the Initial Stage of the 2019 Coronavirus Disease (COVID-19) Epidemic among the General Population in China. Int J Environ Res Public Health.

[11] Brooks SK, Webster RK, Smith LE, Woodland L, Wessely S, Greenberg N, Rubin GJ (2020). The psychological impact of quarantine and how to reduce it: rapid review of the evidence. The Lancet.

[12] Qiu J, Shen B, Zhao M, Wang Z, Xie B, Xu Y (2020). A nationwide survey of psychological distress among Chinese people in the COVID-19 epidemic: implications and policy recommendations. Gen Psych.

[13] Mazza C, Ricci E, Biondi S, Colasanti M, Ferracuti S, Napoli C, Roma P (2020). A Nationwide Survey of Psychological Distress among Italian People during the COVID-19 Pandemic: Immediate Psychological Responses and Associated Factors. Int J Environ Res Public Health.

[14] Gao J, Zheng P, Jia Y, Chen H, Mao Y, Chen S, Wang Y, Fu H, Dai J (2020). Mental health problems and social media exposure during COVID-19 outbreak. PLoS One.

[15] Al-Shamsi HO, Alhazzani W, Alhuraiji A, Coomes EA, Chemaly RF, Almuhanna M, Wolff RA, Ibrahim NK, Chua MLK, Hotte SJ, Meyers BM, Elfiki T, Curigliano G, Eng C, Grothey A, Xie C (2020). A Practical Approach to the Management of Cancer Patients During the Novel Coronavirus Disease 2019 (COVID-19) Pandemic: An International Collaborative Group. Oncologist.

[16] Wang H, Zhang L (2020). Risk of COVID-19 for patients with cancer. The Lancet Oncology.

[17] Yu J, Ouyang W, Chua MLK, Xie C (2020). SARS-CoV-2 Transmission in Patients With Cancer at a Tertiary Care Hospital in Wuhan, China. JAMA Oncol.

[18] Zhang L, Zhu F, Xie L, Wang C, Wang J, Chen R, Jia P, Guan HQ, Peng L, Chen Y, Peng P, Zhang P, Chu Q, Shen Q, Wang Y, Xu SY, Zhao JP, Zhou M (2020). Clinical characteristics of COVID-19-infected cancer patients: a retrospective case study in three hospitals within Wuhan, China. Ann Oncol.

[19] Pellino G, Spinelli A (2020). How Coronavirus Disease 2019 Outbreak Is Impacting Colorectal Cancer Patients in Italy: A Long Shadow Beyond Infection. Dis Colon Rectum.

[20] Kang C, Yang S, Yuan J, Xu L, Zhao X, Yang J (2020). Patients with chronic illness urgently need integrated physical and psychological care during the COVID-19 outbreak. Asian J Psychiatr.

[21] (2020). Clinical management of COVID-19. World Health Organization.

[22] WeChat. Wikipedia.

[23] Zigmond AS, Snaith RP (1983). The hospital anxiety and depression scale. Acta Psychiatr Scand.

[24] Smarr KL, Keefer AL (2011). Measures of depression and depressive symptoms: Beck Depression Inventory-II (BDI-II), Center for Epidemiologic Studies Depression Scale (CES-D), Geriatric Depression Scale (GDS), Hospital Anxiety and Depression Scale (HADS), and Patient Health Questionnaire-9 (PHQ-9). Arthritis Care Res (Hoboken).

[25] Du J, Dong L, Wang T, Yuan C, Fu R, Zhang L, Liu B, Zhang M, Yin Y, Qin J, Bouey J, Zhao M, Li X (2020). Psychological symptoms among frontline healthcare workers during COVID-19 outbreak in Wuhan. Gen Hosp Psychiatry.

[26] Vancampfort D, Firth J, Schuch FB, Rosenbaum S, Mugisha J, Hallgren M, Probst M, Ward PB, Gaughran F, De Hert M, Carvalho AF, Stubbs B (2017). Sedentary behavior and physical activity levels in people with schizophrenia, bipolar disorder and major depressive disorder: a global systematic review and meta-analysis. World Psychiatry.

[27] Cao W, Fang Z, Hou G, Han M, Xu X, Dong J, Zheng J (2020). The psychological impact of the COVID-19 epidemic on college students in China. Psychiatry Res.

[28] Cheng C, Jun H, Liang B (2014). Psychological health diathesis assessment system: a nationwide survey of resilient trait scale for Chinese adults. Stud Psychol Behav.

[29] Huckins JF, daSilva AW, Wang W, Hedlund E, Rogers C, Nepal SK, Wu J, Obuchi M, Murphy EI, Meyer ML, Wagner DD, Holtzheimer PE, Campbell AT (2020). Mental Health and Behavior of College Students During the Early Phases of the COVID-19 Pandemic: Longitudinal Smartphone and Ecological Momentary Assessment Study. J Med Internet Res.

[30] Cohen N, Arieli T (2011). Field research in conflict environments: Methodological challenges and snowball sampling. Journal of Peace Research.

